# Asthma Hot Spots in New York Before and During the COVID-19 Pandemic

**DOI:** 10.5888/pcd21.240059

**Published:** 2024-09-05

**Authors:** Samira Skochko, Trang Nguyen, Stephanie Mack, Brooke Turcotte, Catherine Adler, Eli S. Rosenberg, Christopher Joseph, Lynley Siag, Alexandra Dubuisson, Victoria L. Wagner

**Affiliations:** 1 Public Health Information Group, Office of Science and Technology, New York State Department of Health, Albany; 2 Bureau of Environmental and Occupational Epidemiology, New York State Department of Health, Albany; 3 Office of Science and Technology, New York State Department of Health, Albany; 4 Bureau of Community Chronic Disease Prevention, New York State Department of Health, Menands; 5 Office of Health Services Quality and Analytics, New York State Department of Health, Albany

**Figure:**
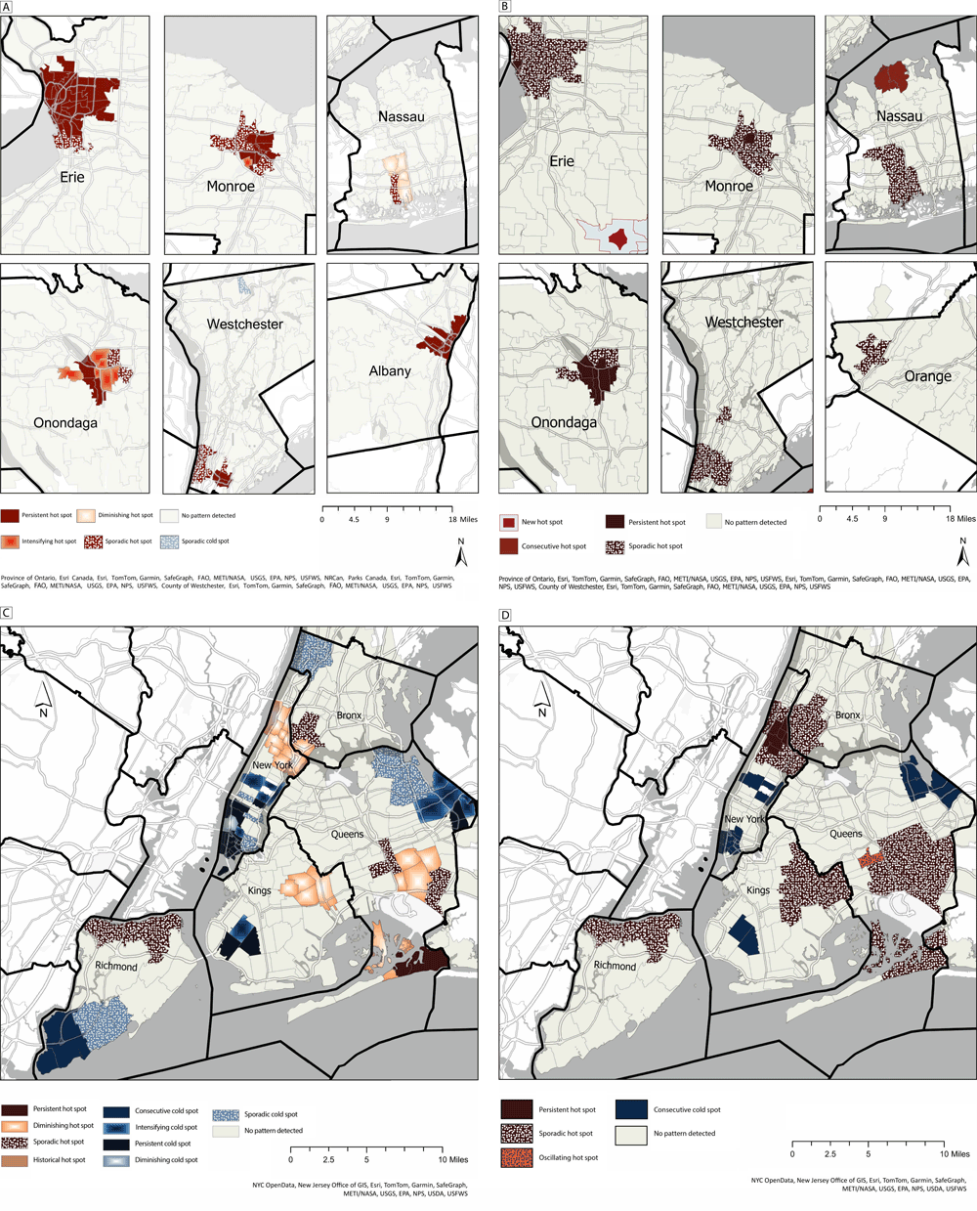
Panels A and B show results of Emerging Hot Spot Analysis (Esri) by zip code level for counties outside New York City with a high asthma burden, before and during the COVID-19 pandemic. Hot spots were detected in urban areas, including Albany (Albany County), Buffalo (Erie County), Rochester (Monroe County), Syracuse (Onondaga County), around Hempstead (Nassau County), and around Yonkers (Westchester County). The analyses for Dutchess and Orange counties did not yield significant results in the 2016 to 2019 time period. The analyses for Dutchess and Albany counties did not yield significant results in the 2020 to quarter 2, 2022 time period. Results for Albany County may be affected by missing data caused by a known lag in reporting by area hospitals during the pandemic. A: Emerging Hot Spot Analysis of zip code-level quarterly asthma emergency department visits per 10,000 population in high-asthma-burden counties, New York State, 2016 to 2019. B: Emerging Hot Spot Analysis of zip code-level quarterly asthma emergency department visits per 10,000 population in high-asthma-burden counties, New York State, 2020 to quarter 2, 2022. Panels C and D show results of Emerging Hot Spot Analysis by zip code level and by county for New York City before and during the COVID-19 pandemic. The dark gray lines represent county borders, and the light gray lines represent zip code borders. The area of zip codes in New York City is shaded according to the emerging hot spot result legend. Panel C shows quarterly asthma emergency department visits per 10,000 population in high asthma burden counties, 2016 to 2019. Panel D shows quarterly emergency department visits per 10,000 population in high asthma burden counties, 2020 to quarter 2, 2022.

## Background

Asthma, a chronic lung disease, is controllable with guidelines-based clinical care and proper self-management (1). The New York State (NYS) Department of Health Asthma Control Program analyzes county and zip code–level emergency department (ED) and hospital discharge data, regularly producing stable 3-year combined estimates that identify high-burden areas for targeted interventions under the NYS Children’s Asthma Initiative (2,3). Since early 2020, routine surveillance has revealed a sharp decline in rates of asthma-related ED visits and hospitalizations. The COVID-19 pandemic severely affected NYS (3,4). Disruptions in care because of limited access to health care facilities and fear of virus transmission likely contributed to the change in rates (5,6).

Traditional mapping of average rates can pinpoint high-burden zip codes in specific time periods. Plotting trends with these copious amounts of data points produces hard-to-interpret visualizations, limiting early identification of emerging areas of concern. The Space Time Pattern Mining approach in ArcGIS Pro’s Emerging Hot Spot Analysis (Esri) analyzes this hard-to-represent information in a 2-dimensional map representation of multidimensional temporal and geographic relationships. To evaluate the practical use of this method, we used it to analyze asthma ED visit data.

## Data and Methods

We obtained all ED visits for NYS residents with a discharge diagnosis of asthma (ICD-10-CM code of J45) (7) from January 1, 2016, through June 30, 2022, from the Statewide Planning and Research Cooperative System and aggregated them by patient residence zip code (8). By using US Census Bureau population estimates, we computed quarterly ED visit rates and joined them to an NYS zip code shapefile in ArcGIS Pro version 3.2.1 (Esri) for analysis. The Space Time Pattern Mining approach in Emerging Hot Spot Analysis assesses patterns by using the Getis-Ord Gi* statistic (Esri) to evaluate clustering of trends evaluated by the Mann-Kendall trend test. The result locates significant (*P* < .10, as specified by the tool) clustering of trends for defined locations and provides a snapshot of trend activity at regular intervals (9,10). We created 2 distinct periods for analyses: prepandemic (2016–2019) and pandemic (2020–June 2022). NYS Children’s Asthma Initiative interventions are prioritized within 13 identified high-burden counties; therefore, we selected county-level analysis (Appendix). We used the Create Space Time Cube from the Defined Locations tool in ArcGIS Pro to form 13 pairs of space–time cubes per county by time period, with bins composed of quarterly zip-code–level asthma-related ED rates (11).

We ran the Emerging Hot Spot Analysis Tool for each cube (12,13) and used a fixed distance for spatial comparisons, calculated by the tool for each cube analyzed. The analysis requires a minimum of 10 time intervals and 30 spatial divisions. Results characterize each zip code into 1 of 17 predefined categories (9). Persistent and intensifying hot or cold spots require a threshold of 90% of all time–space bins per cube meeting significance (9).

## Highlights

Results showed more significant hot spots in NYS urban areas than in nonurban areas for both periods. Overall, ED rates during the pandemic were lower than prepandemic estimates. As opposed to signaling increases or decreases in rates overall, we characterized the variation in local patterns during each time period. We identified prepandemic asthma hot spots for 19 zip codes in Buffalo (Erie County), 12 in Rochester (Monroe County), 11 in Syracuse (Onondaga County), 7 in Albany (Albany County), 6 around Yonkers (Westchester County), and 5 around Hempstead (Nassau County) (Figure, Panel A). Dutchess and Orange Counties had no significant hot or cold spots identified.

During the pandemic, similar hot spot patterns appeared in Buffalo, Rochester, and Syracuse; however, many previously persistent hot spots were later classified as sporadic (Figure, Panel B). Zip code 12746 (Orange County) emerged as a new sporadic hot spot. Westchester hot spots spread to include 9 zip codes classified as sporadic. Hot spots in Nassau County increased to 9 with no cold spots identified. No significant patterns were detected for Dutchess or Albany Counties.

New York City (NYC) showed more hot spots and fewer cold spots during the prepandemic (Figure, Panel C) compared with the pandemic period (Figure, Panel D). The cold spot reduction was observed in northeastern Queens and southern Richmond counties, and an increasing number of hot spots were observed in New York, Bronx, Kings, and Queens counties. In upper New York County, zip codes 10026, 10027, 10030, 10031, 10037, and 10039 were diminishing hotspots prepandemic and became persistent hotspots during the pandemic.

## Action

Traditional analyses are widely used by asthma partners to identify high-burden areas. High ED rates are concentrated around urban areas in NYS, consistent with literature describing the effects of urban factors on asthma (14). Emerging hot spot mapping provides additional tools for assessing and visualizing spatial patterns. However, results should be interpreted with caution and considered in combination with results from traditional analyses.

Hot spot model results may vary depending on the selection of geographic boundaries. Analysis of all zip codes in NYS or NYC or the 2 together, for example, did not yield the same results as individual county-level analysis. Areas known to have the highest rates via traditional methods were not always classified as hot spots (3). Reductions in some quarterly ED rates during the pandemic resulted in greater variation during this time period. The structure of fixed hot spot-result categorization contributed to areas being labeled as undesignated in high-burden counties. For example, the persistent hot spot definition requires that a location be a significant hotspot for 90% of all time-step intervals, a requirement that was not met within the zip code because of fluctuations in quarterly rates, especially during 2020, together with the use of the county boundary and fixed distance bands for geographic clustering criteria (9). For example, zip codes 10453, 10466, and 10467 in Bronx County had several quarterly rates above the maximum values in Queens and Richmond counties, but were not categorized by the tool as hot spots.

Maps produced by using Esri’s Emerging Hot Spot Analysis tool can provide additional insight into patterns of asthma-related health care use in NYS’s high-burden counties. The technique may be applied to other chronic conditions and to identify geographies where socioeconomic inequalities contribute to a disproportionate burden of adverse health outcomes (15). This additional insight will be an important tool in the evaluation of local-level interventions.

## Figures and Tables

**Supplemental Figure:**
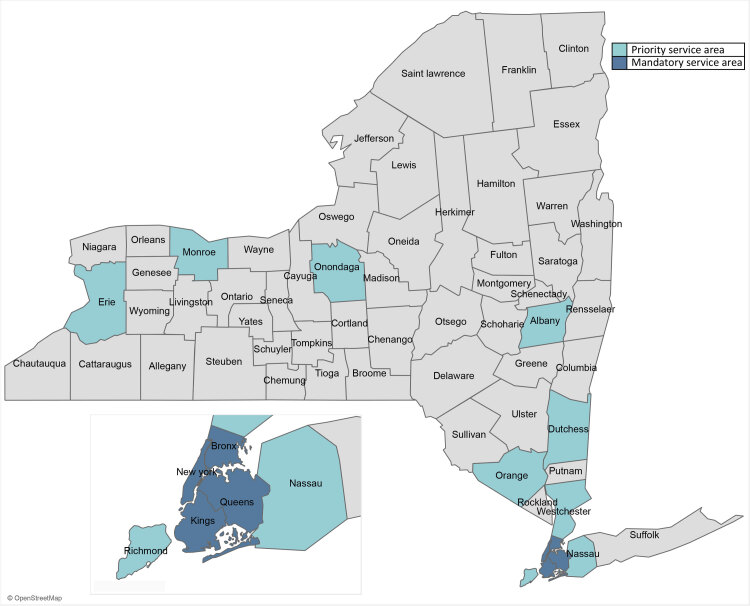
New York State Asthma Control Program service areas in counties with the highest asthma burden. The New York State Children’s Asthma Initiative extends to every county in New York, focusing on mandatory and priority service areas comprising 13 of the highest-burden counties.
